# A validation dataset for Macaque brain MRI segmentation

**DOI:** 10.1016/j.dib.2017.11.008

**Published:** 2017-11-04

**Authors:** Yaël Balbastre, Denis Rivière, Nicolas Souedet, Clara Fischer, Anne-Sophie Hérard, Susannah Williams, Michel E. Vandenberghe, Julien Flament, Romina Aron-Badin, Philippe Hantraye, Jean-François Mangin, Thierry Delzescaux

**Affiliations:** a UMR9199, CNRS, CEA, Paris-Sud Univ., Univ. Paris-Saclay, Fontenay-aux-Roses, France; b MIRCen, Institut de biologie François Jacob, DRF, CEA, Fontenay-aux-Roses, France; c UNATI, NeuroSpin, Institut des sciences du vivant Frédéric Joliot, DRF, CEA, Univ. Paris-Saclay, Gif-sur-Yvette, France; d CATI Multicenter Neuroimaging Platform, France; e US27, INSERM, Fontenay-aux-Roses, France; f Sorbonne Universités, Université Pierre et Marie Curie, Paris, France

## Abstract

Validation data for segmentation algorithms dedicated to preclinical images is fiercely lacking, especially when compared to the large number of databases of Human brain images and segmentations available to the academic community. Not only is such data essential for validating methods, it is also needed for objectively comparing concurrent algorithms and detect promising paths, as segmentation challenges have shown for clinical images.

The dataset we present here is a first step in this direction. It comprises 10 T2-weighted MRIs of healthy adult macaque brains, acquired on a 7 T magnet, along with corresponding manual segmentations into 17 brain anatomic labelled regions spread over 5 hierarchical levels based on a previously published macaque atlas (Calabrese et al., 2015) [1].

By giving access to this unique dataset, we hope to provide a reference needed by the non-human primate imaging community. This dataset was used in an article presenting a new primate brain morphology analysis pipeline, Primatologist (Balbastre et al., 2017) [2]. Data is available through a NITRC repository (https://www.nitrc.org/projects/mircen_macset).

**Specifications Table**

**Table:** 

Subject area	*Biomedical Imaging, Neuroscience*
More specific subject area	*Cynomolgus monkey (Macaca fasciscularis) brain, MRI segmentation*
Type of data	*3D Images (MRI, Manual segmentation)*
How data was acquired	*Animals: Macaca Fascicularis (Noveprim, Mauritus Island)*
	*MRI: 7T MRI scanner (Agilent, Santa Clara, CA, USA)*
	*Segmentation: Cintiq 24HD (Wacom, Saitama, Japan) & Anatomist (NeuroSpin, CEA, France)*
Data format	*NifTi*
Experimental factors	*10 healthy male cynomolgus monkeys aged 3–5 years and weighing 3.8 to 6.3 kg.*
	*Anesthesia during MRI acquisition: ketamine (1 mg/kg), xylazine (0.5 mg/kg), propofol (1 ml/kg/h).*
Experimental features	*T2w Fast Spin Echo (TE: 20 ms, TR: 7500* *ms, 8 averages)*
	*80 coronal slices (thickness: 0.8 mm)*
	*192* × *192 matrix (zero-filling: 256* × *256)*
	*115.2* × *115.2 mm FOV*
Data source location	*Mauritius Island, Republic of Mauritius (animal collection)*
	*Fontenay-aux-Roses, France (housing and imaging)*
	*Gif-sur-Yvette, France (numeric storage)*
Data accessibility	*NITRC:*https://www.nitrc.org/projects/mircen_macset
	*BrainVISA (4.6):*http://www.brainvisa.info

**Value of the data**

• Reference data for algorithms dedicated to the non-human primate brain is lacking.
• This is the first publicly available set of manual segmentations of *Macaca fascicularis* brain MRIs.
• Segmentation into 17 anatomical regions was performed in 15 relevant sections in all three incidences (axial, coronal and sagittal).
• Data is shipped within BrainVISA, along with a process allowing computing section-wise Dice scores.

## 1 Data

MR images of the brain of 10 healthy young adult macaques were acquired on a 7T scanner. In each volume, 15 relevant sections (7 coronal, 5 axial, 3 sagittal, encompassing all the major brain anatomic regions), spanning the whole brain, were selected for manual segmentation. Raw MRIs and manual segmentations are available as NifTi volumes in a NITRC repository (https://www.nitrc.org/projects/mircen_macset). They will also be available with the next release of BrainVISA (version 4.6, http://www.brainvisa.info), through the BrainVISA installer. This dataset is distributed under the CeCILLv2.1 license (http://www.cecill.info), a GPL-compatible license, and can be freely used for academic work, upon citing this paper. This dataset was used to validate automated segmentations obtained with Primatologist, a pipeline dedicated to macaque brain morphology analysis [2]. The list of provided files is summarized in Table 1.

**Table 1:** Files comprised in the dataset. Subjects are named M01 to M10.

**Filename**	**Description**
MAC[*].nii.gz	Raw T2-weighted MRI.
MAC[*]_manual_axial.nii.gz	Manual segmentation of 5 axial sections.
MAC[*]_manual_coronal.nii.gz	Manual segmentation of 7 coronal sections.
MAC[*]_manual_sagittal.nii.gz	Manual segmentation of 3 sagittal sections.
MAC[*]_manual_merged.nii.gz	Fusion of manual segmentations in all three incidences.
MAC[*]_manual_[*]_mask.nii.gz	Mask of the manually segmented sections. It must be used to compute F_1_ scores.
hierarchy.csv/.hie	Ontology and labels associated with the segmented regions.
classificationScores.py	Set of python functions allowing computing classification scores.

## 2 Experimental design, materials and methods

### 2.1 Animals and imaging

All animal studies were conducted according to European regulations (EU Directive 2010/63) and in compliance with Standards for Humane Care and Use of Laboratory Animals of the Office of Laboratory Animal Welfare (OLAW – no#A5826-01) in a facility authorized by local authorities (authorization no#B92-032-02). All efforts were made to minimize animal suffering and animal care was supervised by veterinarians and animal technicians skilled in the healthcare and housing of NHPs. All animals were housed under standard environmental conditions (12-h light-dark cycle, temperature: 22 ± 1 °C and humidity: 50%) with ad libitum access to food and water.

Ten male cynomolgus monkeys (*Macaca fascicularis*, supplied by Noveprim, Mauritius Island) aged 2 to 5 years (mean: 3.76, SD: 0.77) underwent baseline MR imaging. Animals weighed 3.8 to 6.3 kg (mean: 4.85, SD: 0.73) at examination time (see Table 2 for detailed age and weight). Animals were anesthetized with ketamine (1 mg/kg) and xylazine (0.5 mg/kg), maintained with intravenous infusions of propofol (1 ml/kg/h) and placed in the magnet in a sphinx position with the head fixed in a stereotaxic MRI-compatible frame (M2E, France). Animals were heated by a hot air flux and their temperature and respiration parameters were monitored remotely.

**Table 2:** Subject age and weight at the MRI scan time.

**Macaque identity**	**Age (years)**	**Weight (kg)**
MAC01	2.20	3.84
MAC02	2.73	4.20
MAC03	3.93	4.50
MAC04	3.91	4.97
MAC05	3.96	4.10
MAC06	3.92	5.20
MAC07	3.94	4.73
MAC08	3.98	6.30
MAC09	3.99	5.30
MAC10	5.01	5.34

Eighty coronal slices (thickness: 0.8 mm) were acquired using a 7 T MRI scanner (Agilent, Santa Clara, CA, USA) with a T2w Fast Spin Echo sequence (TE: 20 ms, TR: 7500 ms, 8 averages, acquisition time: 48 min) on a 192 × 192 matrix with a 115.2 × 115.2 mm field of view, yielding a 0.6 × 0.6 × 0.8 mm^3^/voxel resolution. The K -space was zero-filled to a 256 × 256 × 80 matrix for a final voxel size of 0.45 × 0.45 × 0.8 mm^3^.

### 2.2 Preprocessing

Data format was converted with BrainVISA from Varian FDF, produced by the scanner, to gzipped NifTi. Because Varian's on-disk storage is sequence-dependent and does not provide voxel-to-world transforms, volumes were flipped, and the appropriate voxel-to-world transforms were stored into the NifTi header, with BrainVISA's python tools (pyAIMS) so that volumes can be used with any software able to handle NifTi metadata. Offsets were set so that the origin is the center of the volume. See https://nifti.nimh.nih.gov for additional precisions on the NifTi format and the way it handles transforms.

### 2.3 Regions definition

**Fig. 1:**
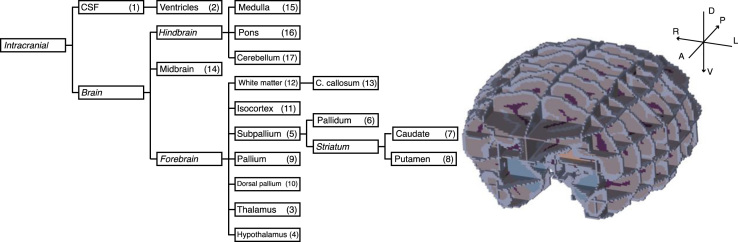
Left: simplified CIVM hierarchy. Only labels associated with a number correspond to a hard label. The others (in italics) are built by aggregation and can be used for multi-scale evaluation. Right: Manual segmentation of a representative subject: 7 coronal, 5 axial and 3 sagittal sections spanning the entire brain were segmented. The dorso-ventral (D-V), antero-posterior (A-P) and left-right (L-R) axes are also depicted.

The choice of regions to segment was based on the rhesus macaque atlas published by the Center for in vivo Microscopy (CIVM) [1]. This atlas consists in the segmentation into 241 regions of a template (i.e., a mean image) built from MRIs acquired in 10 *post mortem* brain specimen. These regions belong to an ontology that we used to simplify the atlas and reduce it to 17 major labels and 5 hierarchical levels. The resulting simplified ontology is shown in Fig. 1, along with the corresponding numeric labels.

### 2.4 Sections selection

Rather than manually segmenting all 80 coronal slices that constitute a MR volume, we decided to select a subset of relevant sections in all three incidences. This choice was guided by the will to avoid any incidence-induced bias in the segmentation as well as lower the segmentation load. As a result, 7 coronal, 5 axial and 3 sagittal sections were selected so that all anatomical classes were found in all three incidences.

Coronal sections were selected based on the Paxinos Macaque Atlas [3] relatively to the anterior commissure (AC) and posterior commissure (PC). These coordinates were then converted to pseudo-Talairach coordinates (without alignment of the AC-PC axis). Let us note AL the brain anterior limit and PL the brain posterior limit. Selected coordinates are provided in Table 3. A rough correspondence with sections from the CIVM template is also provided.

**Table 3:** Coordinates of the 7 selected coronal sections in Paxinos, Talairach and CIVM referentials.

**Paxinos Bregma**	**Paxinos AC–PC**	**Pseudo-Talairach**	**CIVM**
Bregma + 10.85 mm	AC + 14 mm	AC + 0.51 × (AL − AC)	134
Bregma + 3.85 mm	AC + 7 mm	AC + 0.26 × (AL − AC)	180
Bregma − 3.15 mm	AC	AC	227
Bregma − 10.125 mm	0.5 × (AC + PC)	0.5 × (AC − PC)	274
Bregma − 17.1 mm	PC	PC	320
Bregma − 24.1 mm	PC – 7 mm	PC – 0.15 × (PC − PL)	367
Bregma − 31.1 mm	PC – 14 mm	PC – 0.3 × (PC − PL)	413

Axial sections were selected based on the CIVM atlas. Let us note DL the brain dorsal limit, VL the brain ventral limit and *C* = 1/2 (AC + PC) the brain central point along the dorso-ventral axis. Selected sections were: *C*+2/3 (DL–*C*), *C*+1/3 (DL–*C*), *C*, *C*−1/3 (*C*–VL), *C*–2/3 (*C*–VL).

Sagittal sections were also selected based on the CIVM atlas. Let us note LL the brain left limit, RL the brain right limit and IHP the interhemispheric plane. Selected sections were: IHP+1/2 (LL-IHP), IHP, IHP−1/2 (IHP-RL).

### 2.5 Manual segmentation

A single operator manually segmented the 17 anatomical regions present in the simplified atlas with a Cintiq 24HD touchscreen (Wacom, Saitama, Japan), using the Anatomist software (NeuroSpin, CEA, France). Both the CIVM and the Paxinos atlases were used as references to delineate the structures. A representative example is shown in Fig. 1.

Segmentations were stored in Anatomist's ARG format in three different files per animal (one per incidence), and then converted to NifTi. The voxel-to-world transform of the raw MRI was also stored in the segmentation NifTi files. A 3D segmentation volume was also created by fusing the three incidences. A majority vote was used to fill overlapping voxels.

### 2.6 Classification scores

This dataset is provided with a set of python functions, which allow computing classification scores with our manual segmentations as a ground truth. Because scores can only be based on sections that were manually segmented, the corresponding sections in the evaluated 3D segmentations can be automatically extracted.

Image segmentation can be seen as a classification problem, a domain where the *F*_1_ score is a widely used metric. The *F*_1_ score is exactly equivalent to the Dice coefficient [4], a more common designation in the field of image segmentation. For a given class, let us call *P* the set of voxels that belong to it and *F* the set of non-belonging voxels. Then |*P*|+|*F*| = *n*, the number of voxels in the image. Let us note *C*_P_ the set of voxels classified as belonging to the class and *C*_F_ those classified as non-belonging. True positives (TPs) are voxels accurately classified as belonging to the region (*C*_P_ ∩ *P*) and false positives those inaccurately classified as belonging to the region (*C*_P_ ∩ *F*). True negatives (*C*_F_ ∩ *F*) and false negatives (*C*_F_ ∩ *P*) are defined the same way.

Precision is defined as the ratio between the number of TPs and the number of observations classified as positive and can be seen as a measure of over-segmentation:

$$p=\frac{\left|C_{P}⋂P\right|}{\left|C_{P}\right|}$$

Precision vary between 0 and 1, with a maximal score indicating no type I error, i.e. no over-detection. Recall is defined as the ratio between the number of TPs and the number of truly positive observations and can be seen as a measure of under-segmentation:

$$r=\frac{\left|C_{P}⋂P\right|}{\left|P\right|}$$

Recall varies between 0 and 1, with a maximal score indicating no type II error, i.e. no under-detection. The *F*_1_ score is defined as the harmonic mean of precision and recall and thus includes information on both over- and under-segmentation:

$$f_{1}=\frac{p∙r}{p+r}$$

Consequently, the *F*_1_ score also vary between 0 and 1, with higher scores indicating agreement between segmentations.

However, this score was only defined for binary classifications, where observations can be separated between positive and negative. In the case of multi-labels segmentation, it must be extended. We used the micro-averaged *F*_1_ with a multi-labels definition of sets *P* (positives) and *C*_P_ (classified as positives), according to the conventional *F*_1_ formula. Let *R* the ground truth volume and *S* the evaluated segmentation:

$$P=\left(\left\{i,R_{i}\right\};\;\text{if}\;R_{i}>0\right)$$

$$C_{P}=\left(\left\{i,S_{i}\right\};\;\text{if}\;S_{i}>0\right)$$

The provided set of functions allows computing the binary *F*_1_ score for each node of the atlas hierarchy, as well as the micro-*F*_1_ score. User-friendly processes will also be available within the next release of BrainVISA (4.6).
